# Fluctuations in Affective States and Self-Efficacy to Resist Non-Suicidal Self-Injury as Real-Time Predictors of Non-Suicidal Self-Injurious Thoughts and Behaviors

**DOI:** 10.3389/fpsyt.2020.00214

**Published:** 2020-03-20

**Authors:** Glenn Kiekens, Penelope Hasking, Matthew K. Nock, Mark Boyes, Olivia Kirtley, Ronny Bruffaerts, Inez Myin-Germeys, Laurence Claes

**Affiliations:** ^1^School of Psychology, Curtin University, Perth, WA, Australia; ^2^Department of Neurosciences, Center for Public Health Psychiatry, Leuven, Belgium; ^3^Department of Psychology, Harvard University, Cambridge, MA, United States; ^4^Department of Neurosciences, Center for Contextual Psychiatry, KU Leuven, Leuven, Belgium; ^5^Institute for Social Research, Population Studies Center, University of Michigan, Ann Arbor, MI, United States; ^6^Faculty of Psychology and Educational Sciences, KU Leuven, Leuven, Belgium; ^7^Faculty of Medicine and Health Sciences (CAPRI), University of Antwerp, Antwerp, Belgium

**Keywords:** non-suicidal self-injury, real-time prediction, ideation-to-action, intensive longitudinal assessment, ecological momentary assessment

## Abstract

**Introduction:**

Although research over the past decade has resulted in significantly increased knowledge about distal risk factors for non-suicidal self-injury (NSSI), little is known about short-term (proximal) factors that predict NSSI thoughts and behaviors. Drawing on contemporaneous theories of NSSI, as well as the concept of ideation-to-action, the present study clarifies (a) real-time factors that predict NSSI thoughts and (b) the extent to which theoretically important momentary factors (i.e., negative affect, positive affect, and self-efficacy to resist NSSI) predict NSSI behavior in daily life, beyond NSSI thoughts.

**Methods:**

Using experience sampling methodology, intensive longitudinal data was obtained from 30 young adults with frequent NSSI episodes in the last year. Participants completed assessments up to eight times per day for 12 consecutive days (signal-contingent sampling). This resulted in the collection of 2,222 assessments (median compliance = 79.2%) during which 591 NSSI thoughts and 270 NSSI behaviors were recorded. Using the dynamic structural equation modeling framework, multilevel vector autoregressive models were constructed.

**Results:**

Within the same assessment, negative affect was positively associated with NSSI thoughts, whereas positive affect and self-efficacy to resist NSSI were each negatively associated with NSSI thoughts. Across assessments, higher-than-usual negative affect and self-efficacy to resist NSSI were predictive of short-term change in NSSI thoughts. While fluctuations in both negative affect and positive affect prospectively predicted NSSI behavior, these factors became non-significant in models that controlled for the predictive effect of NSSI thoughts. In contrast, self-efficacy to resist NSSI incrementally predicted a lower probability of engaging in NSSI, above and beyond NSSI thoughts.

**Discussion:**

This study provides preliminary evidence that affective fluctuations may uniquely predict NSSI thoughts but not NSSI behaviors, and point to the role of personal belief in the ability to resist NSSI in preventing NSSI behavior. These findings illustrate the need to differentiate between the development of NSSI thoughts and the progression from NSSI thoughts to behavior, as these are likely distinct processes, with different predictors.

## Introduction

Non-suicidal self-injury (NSSI), defined as the deliberate, self-inflicted damage of one's own body tissue without suicidal intent (e.g., cutting, scratching, and hitting oneself), is a worrisome behavior among adolescents and emerging adults (1, 2). Pooled lifetime prevalence estimates of NSSI are close to 17%–18% among adolescents and 12%–20% among emerging adults (3, 4). NSSI behaviors are an important predictor of future suicidal thoughts and behaviors (5–8) and psychopathology (9, 10), and are associated with stigma and feelings of shame (11–13), low levels of disclosure and help-seeking (14–16), and other adverse outcomes [e.g., poorer academic performance; (17)]. Together, these findings underscore the importance of a good understanding of the factors that underlie NSSI, with a view to informing preventive and intervention initiatives.

### The Short-Term Prediction Problem in Existing Research on NSSI

While NSSI and its correlates have traditionally been studied using cross-sectional designs, over the past decade, concerted efforts have been made to clarify long-term (distal) predictors (18–21). These longitudinal studies typically take a population-level nomothetic approach (i.e., risk stratification at the between-person level), involving few measurement occasions (usually 2–5) that are spaced over long observation windows (e.g., yearly). Although such an approach may be useful in revealing *who*—within the entire population—is at relatively high risk of engaging in NSSI in the next months or years, it lacks temporal resolution to reliably tell us *when* individuals at high risk are most likely to contemplate, or engage in, NSSI in the next minutes and hours. Providing greater clarity regarding short-term (proximal) predictors requires a specific research design that takes an individualized ideographic approach (i.e., risk stratification at the within-person level) as well as intensive monitoring to capture momentary processes that explain imminent risk of NSSI. Fortunately, the recent proliferation of new technologies and smartphone-based apps have now made it feasible to use experience sampling methods to study NSSI and its real-time predictors in daily life (22).

### Affective Disturbances and NSSI

A central focus of most theoretical models is that NSSI most often serves an affect regulation function (23–26). Empirical work supports that affect regulation is the most common reported reason for NSSI (27), and, consequently, many studies have focused on the predictive value of affective traits at the between-person level (28). This work revealed that both higher trait negative affect (i.e., tendency to experience more negative emotions) and lower trait positive affect (i.e., tendency to experience less positive emotions) are associated with risk of lifetime (29–31) and future NSSI behavior (32, 33). In line with this, emotional problems (especially anxiety and depressive symptoms) have been identified as risk factors of NSSI at the between-person level (18, 19, 34). Unfortunately, fewer studies have focused on the role of affect in determining short-term risk for NSSI at the within-person level [for an overview see (35)]. One consistent finding across studies is that negative affect increases prior to NSSI (36–38), and predicts a higher probability of NSSI in the next hours (39–42). For instance, using ecological momentary assessment, Kranzler and colleagues observed that a momentary increase in negative affect positively predicted NSSI in the following 2–3 hours for adolescents and young adults (40). Similarly, Houben and colleagues, demonstrated that higher-than-usual negative affect increased the likelihood of NSSI in the next 30–120 minutes among a sample of inpatients (39). While these findings are important and support the affect regulation function of NSSI, future work is required beyond these initial studies to clarify the specificity of affective fluctuations in the short-term prediction of NSSI at the within-person level.

Of note, more research is warranted investigating the potential utility of positive affect as a protective factor against NSSI, as evidence to date has yielded inconclusive results. While some researchers have observed a decrease in positive affect in the hours prior to engagement in NSSI (36, 37), others failed to confirm such a time trend (38), and found that lower-than-usual positive affect is not prospectively predictive of NSSI (39, 40). It may be that momentary lowered positive affect is more tolerable than increased negative affect, and therefore less relevant in eliciting NSSI (40). In any case, better understanding the role of affect requires thorough examination of specific emotions (e.g., relaxed, stressed), as well as composite constructs (e.g., positive affect). Retrospective studies, for instance, have demonstrated that people who self-injure report increased levels of positive emotions low in arousal (e.g., satisfied, relaxed) as well as decreased levels of negative emotions high in arousal (e.g., anxious, stressed) from prior to post NSSI (43, 44). However, because these studies are susceptible to memory biases that may distort these findings, experience sampling studies are warranted to evaluate whether low-arousal positive emotions, and/or high-arousal negative emotions, are most relevant in predicting NSSI within the next few hours. Providing greater clarity regarding the specificity of affective states as short-term predictors of NSSI would provide valuable information for the development of novel preventive interventions.

### Affective Disturbances Predictive of NSSI Thoughts or NSSI Behavior?

Surprisingly little is known about the extent to which affective fluctuations predict NSSI behavior, beyond NSSI thoughts. Originating from studies on suicidal thoughts and behaviors (45–47), the ideation-to-action framework argues that the factors that lead people to contemplate about a behavior (i.e., in this case thoughts concerning suicide) may not necessarily be the same factors that govern whether people act on their thoughts (i.e., attempt suicide). In a similar vein, it may be equally important to differentiate between the process of developing NSSI thoughts and engaging in NSSI behavior. NSSI thoughts are an important near-term precursor of NSSI behavior (41, 42), and a growing body of evidence suggests that momentary increased negative affect and lowered positive affect are salient factors in predicting NSSI thoughts (40, 41, 48). As such, similar to the observation that affective disturbances are robust predictors of suicide ideation but not attempt (47, 49–51), the possibility exists that affective fluctuations are relevant in predicting short-term change in NSSI thoughts but are not uniquely predictive of making the transition from NSSI thoughts to behavior. While emerging evidence suggests that fluctuations in positive affect might be more useful in predicting thoughts than behavior (40), it is currently unclear whether negative and positive affective states hold predictive value beyond NSSI thoughts in determining short-term risk of NSSI behavior. Addressing this important gap in knowledge requires that future experience sampling studies carefully consider NSSI thoughts when evaluating affective states in the prediction of NSSI behavior.

If affective fluctuations are more useful in explaining short-term change in NSSI thoughts than in predicting the occurrence of NSSI behavior, an important question is whether we can identify momentary factors that provide added insight into whether someone will transition from NSSI thoughts to behavior. Contemporaneous models of NSSI have begun to incorporate cognitive processes in explaining when people are at heightened risk of engaging in NSSI (23, 26). According to the Cognitive-Emotional Model of NSSI (23), NSSI-related cognitions determine whether someone who is experiencing an aversive emotional situation will, or will not, engage in NSSI in the next minutes and hours. Specifically, this model postulates that personal belief in the ability to resist NSSI will be a unique protective factor against NSSI behavior. While findings confirm that people who engage in NSSI report lower self-efficacy to resist NSSI than peers who do not self-injure (23, 52, 53), experience sampling studies are warranted to evaluate whether these beliefs have utility in determining risk of NSSI behavior.

### The Present Study

**Figure 1:**
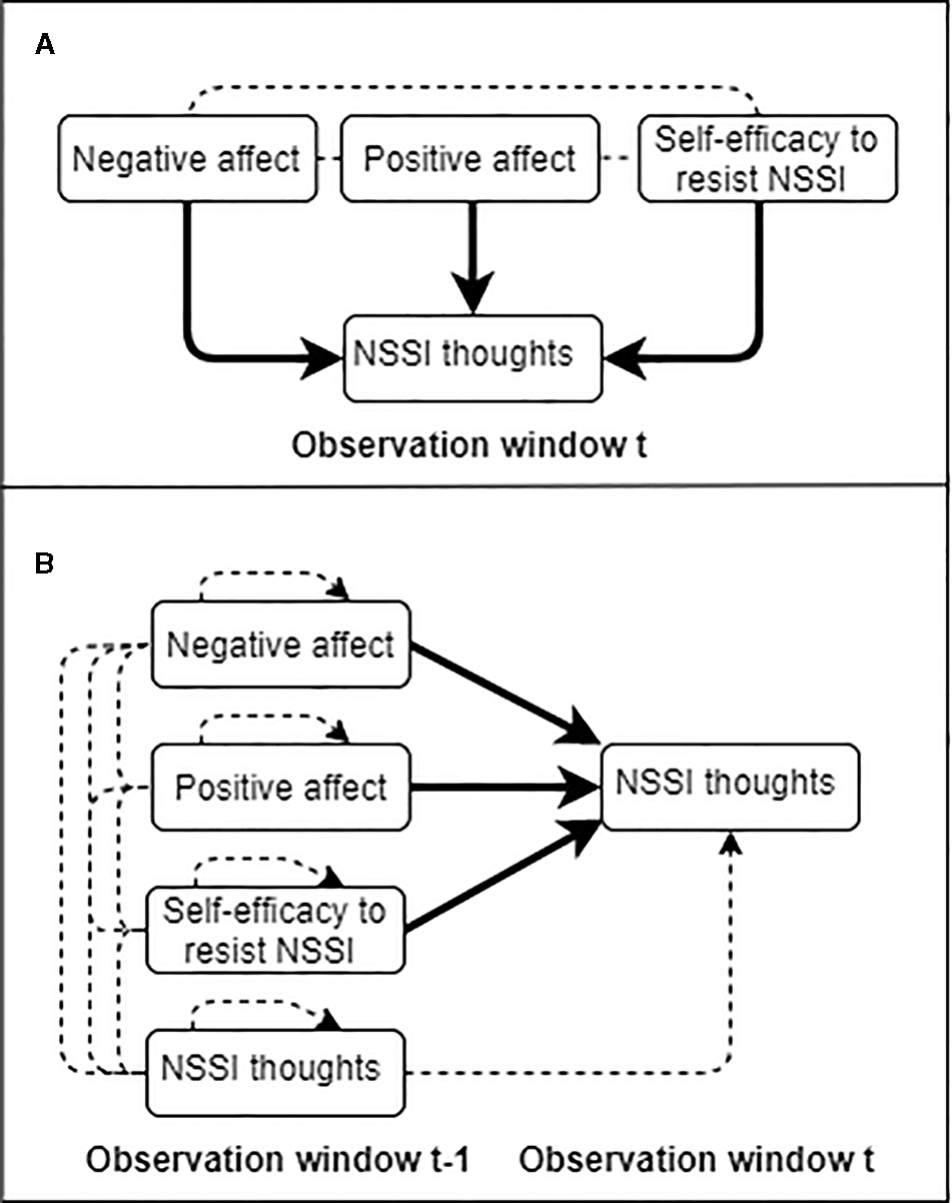
Graphical illustration of the first research objective of the study in which NSSI thoughts are predicted within (Objective 1a depicted in panel **A**) and across measurement windows (Objective 1b depicted in panel **B**) at the within-person level. Solid arrows indicate effects of interest. Dotted arrows represent autoregressive effects and dotted lines associations within the same observation window.

We designed the present study to clarify the extent to which momentary fluctuations in affective states and self-efficacy to resist NSSI are real-time predictors of NSSI thoughts and behaviors. Specifically, there were two major objectives at the within-person level. The first main objective was to evaluate whether within-person fluctuations in negative affect, positive affect, and self-efficacy to resist NSSI predict NSSI thoughts within the same observation window (i.e., contemporaneous associations reflecting processes occurring in the moment; objective 1a in **Figure 1**), as well as from one observation window to the next (i.e., temporal associations reflecting processes that unfold within hours; objective 1b in **Figure 1**). Based on existing knowledge (40, 41, 48), we hypothesized that higher-than-usual negative affect, and lower-than-usual positive affect, would each be contemporaneously and temporally associated with NSSI thoughts. However, as we expected that momentary fluctuations in affective states would trigger NSSI thoughts more strongly within minutes than hours, stronger effects were anticipated in contemporaneous than temporal models (54).

**Figure 2:**
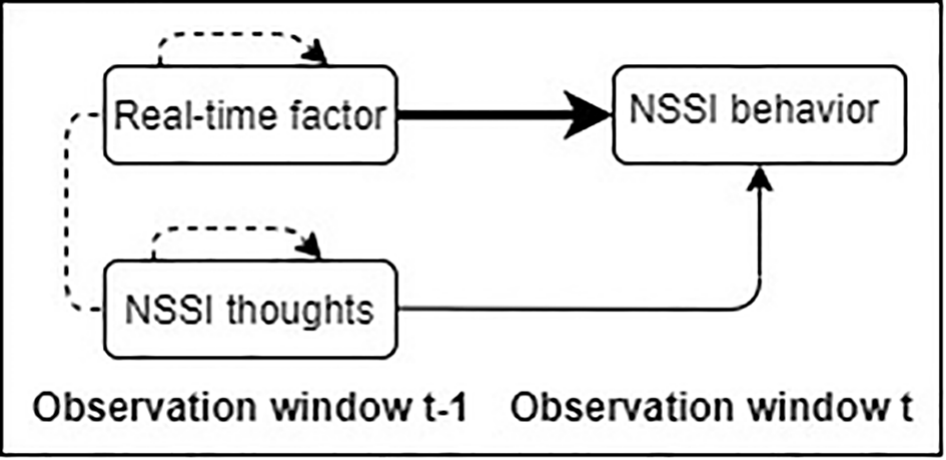
Graphical illustration of the second research objective of the study in which NSSI behavior in daily life is predicted by real-time factors (i.e., negative affect, positive affect, and self-efficacy to resist NSSI), beyond NSSI thoughts at the within-person level. Solid arrows indicate effects of interest. Dotted arrows represent autoregressive effects and dotted lines associations within the same observation window.

The second main objective was to evaluate whether within-person variation in affective states and self-efficacy to resist NSSI, relative to their own average levels, predict NSSI behavior above and beyond NSSI thoughts (**Figure 2**). Building upon previous research from the suicide literature (47, 49, 50), we hypothesized that fluctuations in affective states would not further increase the risk for NSSI behavior, after accounting for NSSI thoughts. To explore the utility of specific emotions, results were also analyzed using emotions as units of analyses rather than composite measures of negative and positive affect. As suggested by the Cognitive-Emotion Model of NSSI (23), we expected that self-efficacy to resist NSSI would negatively predict the occurrence of NSSI behavior above and beyond NSSI thoughts. Finally, in keeping with empirical work at the between-person level (18, 19, 32–34), an additional aim of the study was to evaluate trait negative affect, trait positive affect, self-efficacy to resist NSSI, and anxiety and depressive symptoms assessed at baseline as prospective predictors of NSSI thoughts and behaviors (Objective 3). Consistent with previous research and the ideation-to-action framework (18, 19, 47, 49, 50), we hypothesized that depressive symptoms would uniquely predict mean-level of NSSI thoughts over time but not probability of NSSI behavior, whereas the opposite pattern of results was expected for self-efficacy to resist NSSI.

## Methods

### Participants and Procedure

Participants were 30 young adults (Mean age = 20.1, SD = 1.1; 80.0% female) selected from an ongoing longitudinal survey study of college students (19), meeting inclusion criteria of: (a) NSSI on 5 or more days in the last year, and (b) reported urges to self-injure in the past month. Participants were invited to the laboratory to complete self-report questionnaires and a diagnostic interview, and to receive training on completing the experience sampling protocol *via* “ExpiWell,” a widely used smartphone app for real-time, and real-world, data collection (https://app.expiwell.com). The presence of 12-month mental disorders was assessed by means of the Structured Clinical Interview for DSM-5 (55). A clinical psychologist administered the following sections: mood disorders, anxiety disorders, obsessive-compulsive and related-disorders, substance use disorders, externalizing disorders, post-traumatic stress-disorder, and eating disorders. NSSI characteristics and suicidal thoughts and behaviors were assessed with the Self-Injurious Thoughts and Behaviors Interview (56). **Table 1** presents an overview of the diagnostic features of the sample. Participants met diagnostic criteria for a median of two mental disorders in the last 12 months, with DSM-5 anxiety and mood disorders being most prevalent (range 33.3%–50%; **Table 1**). Participants reported a median of NSSI on 17.5 days in the past year (range 5–360 days), and used a median of 5 NSSI methods (ranging between 2 and 10). Two thirds of the sample (66.7%) also reported having suicidal thoughts in the preceding 12 months, and 20% reported having made at least one suicide attempt in their life (median = 2.5 attempts).

**Table 1:** Diagnostic characteristics of the sample (n = 30).

	%/Median (min; max)
Mood disorders	33.3
Major depressive disorder	26.7
Persistent depressive disorder	20.0
Anxiety disorders	50.0
Panic disorder	13.3
Agoraphobia	6.7
Specific phobia	16.7
Social anxiety disorder	20.0
Generalized anxiety disorder	23.3
Obsessive-compulsive disorders	16.7
Alcohol use disorder (mild-moderate)	13.3
ADHD	6.7
Posttraumatic stress disorder	13.3
Eating disorders	20.0
Anorexia nervosa	13.3
Bulimia nervosa	3.3
Binge-eating disorder	3.3
Any current mental disorder	70.0
Number of current disorders	2.0 (0; 7)
Number of days NSSI past year	17.5 (5; 360)
Number of acts past month	2.0 (0; 60)
Number of methods	5 (2; 10)
Top 3 reported NSSI behaviors	
Scratched oneself	86.7
Cut oneself	66.7
Hit hand/foot against wall/other objects	56.7
Medically treated for NSSI	26.7
Suicidal thoughts and behaviors 12-month suicide ideation	66.7
Lifetime suicide attempt	20.0

Mental disorders were defined as having met diagnostic criteria within the past year, with the exception of generalized anxiety disorder and ADHD which were defined as having met diagnostic criteria within the past 6 months.

ADHD, attention-deficit/hyperactivity disorder; NSSI, non-suicidal self-injury.

Following initial screening, participants were enrolled in a 12-day signal-contingent experience sampling protocol in which they were prompted randomly eight times per day, between 9 a.m. and 9 p.m., in blocks of 1.5-h segments (minimum 15 minutes apart from prior assessments). Participants without a smartphone were provided with a personal device by the research team. To ensure that we captured people in their ongoing activities, and to avoid retrospective reporting, participants were required to register their response within 15 minutes of each prompt. Reimbursement for participation was structured to encourage compliance (42), with a financial compensation of €25 if compliance ranged between 25% and 50%, €50 between 50% and 85%, and €75 if compliance exceeded 85%. Overall compliance was good (median = 79.2%) with, on average, 74 randomly registered recordings per participant (range 36–95), resulting in 2,222 randomly registered recordings for the entire sample. Prior to enrollment, all participants were briefed about the procedures and the voluntary and confidential nature of the study and were provided with contact details of responsible clinicians (including the first and last author, both clinical psychologists). One item of the experience sampling protocol also assessed suicide ideation and activated a popup screen within the app with additional resources when participants reported experiencing suicidal thoughts. Written informed consent was provided by all participants and the study's protocol was approved by the University's Ethical Review Board and by the Belgian commission for the protection of privacy. All procedures were in accordance with the 1964 Helsinki declaration and its later amendments.

### Laboratory Measures

Trait Positive and Negative Affect was assessed using the Positive and Negative Affect Schedule [PANAS; (57)]. The PANAS presents 10 positive (e.g., excited, attentive) and 10 negative emotions (e.g., distressed, nervous), and participants were asked to rate the extent to which they “generally” experience each emotion on a five-point Likert scale ranging from 1 (very slightly or not at all) to 5 (extremely). The PANAS is a reliable and valid measure that is invariant across demographic variables (58), and the internal consistency coefficients of both scales were also good in the current sample (α_PA_ = 0.78, α_NA_ = 0.88).

Self-Efficacy to Resist NSSI was assessed using the six‐item measure adapted from the Self‐Efficacy to Avoid Suicidal Action Scale (59). In this study, participants reported from 1 (very uncertain) to 10 (very certain) whether they believe they can resist engaging in NSSI in the next 2 weeks (e.g., “How certain are you that you will not self‐injure in the next two weeks?”), with higher scores indicating a higher personal belief in the ability to resist NSSI. The adapted NSSI version has shown good internal consistency coefficients in previous research (52, 53) as well as the current sample (α = 0.74).

Anxiety and Depressive Symptoms in the past week were measured using the 21-item Depression Anxiety Stress Scales (60). The anxiety (e.g., “I felt I scared without any good reason”) and depression (e.g., “I felt down-hearted and blue”) scales include seven 4-point items ranging from 1 (Did not apply to me at all) to 4 (Applied to me very much or most of the time) that assess symptoms in the preceding week. Both scales have demonstrated good internal consistency and convergent and discriminant validity in previous research (60). The internal consistency of the scales in the current sample was acceptable for the Anxiety scale (α = 0.61) and good for the Depressive scale (α = 0.89).

### Ecological Momentary Assessment

Momentary Positive and Negative Affect was assessed by asking respondents at each prompt to what extent they currently experience four positive (i.e., excited, cheerful, satisfied, relaxed), and six negative emotions (i.e., stressed, irritated, anxious, sad, hopeless, insecure): “Right now, I feel [emotion].” These specific emotions were selected because they represent a conceptual range of emotions within all quadrants of the affective circumplex defined by the dimensions of valence and arousal (61). Each emotion was rated on a 7-point Likert scale ranging from 0 (not at all) to 6 (very much), with the order in which emotions were presented randomized within persons, across beeps. Each affective state was calculated as a weighted mean across items. Using methods described by Shrout and Lane (62), both scales demonstrated excellent between-person reliability (RKR_PA_ = 0.98, RKR_NA_ = 0.99), and good within-person reliability (RC_PA_ = 0.83, RC_NA_ = 0.77).

#### Momentary NSSI Thoughts and Occurrence of NSSI Behavior

At each prompt, participants were asked to indicate whether they were currently thinking of engaging in NSSI (“Right now, I think about self-injuring without suicidal intent”) using a 7-point Likert scale ranging from 0 (not at all) to 6 (a lot). Additionally, participants were asked to indicate whether or not they engaged in NSSI since their last registration (“Have you self-injured without wanting to die since the last beep?”). If answered affirmatively, a list of NSSI behaviors was presented including cutting/carving, scratching, hitting, burning, biting, head-banging, wound interfering, and an “other” category.

Momentary Self-Efficacy to Resist NSSI was measured by asking participants how confident they felt in their ability to resist NSSI (“How confident are you that you will not engage in NSSI till the next beep”) using a 7-point Likert scale ranging from 0 (not confident at all) to 6 (very much confident).

### Statistical Analyses

To accommodate the two-level structure of the data (i.e., observations nested within persons), and to provide understanding of the value of real-time predictors of NSSI thoughts and behavior, multilevel vector autoregressive models were constructed within the Dynamic Structural Equation Modeling Framework (DSEM) in Mplus 8.3 (63, 64). Contemporaneous associations between factors of interest and NSSI thoughts within the same window of measurement (Objective 1a) were examined using Residual DSEM, which is closely related to the regular DSEM framework, but allows modeling of the autoregressive part of the model while preserving the structural part on the contemporaneous relationships (65). Temporal relationships between factors of interest and both NSSI thoughts and behavior (Objectives 1b and 2) were examined using regular DSEM. This allowed us to investigate the extent to which time-varying variables at *t* − 1 (e.g., negative affect) predict NSSI thoughts and NSSI behavior at *t*, above and beyond the lagged version of the outcome variable (i.e., the autoregressive parameter) and/or a confound variable at *t* − 1 (e.g., NSSI thoughts in the prediction of NSSI behavior). Latent person-mean centering was used to allow interpretation of predictor variables at the within-person level in a relative fashion for each person while accounting for sampling error. At the between-person level, we considered trait negative affect, trait positive affect, baseline self-efficacy, and anxiety and depressive symptoms in the past week as prospective predictors of NSSI thoughts and NSSI behaviors during the 12-day experience sampling protocol (Objective 3). These between-person variables were grand-mean centered to allow interpretation relative to the overall sample mean.

In all models, we used Bayesian estimation based on Markov Chain Monte Carlo using Gibbs sampling. Bayesian estimation has several advantages over a frequentist approach in this context, such as better performance in small samples (i.e., posterior distributions are not required to have asymptotically normal distributions). Non-informative priors were used in all analyses. Point estimates were obtained by taking the median of the posterior distributions for each parameter. Statistical significance was determined by estimating a 95% credibility interval (CI) around each point estimate. A 1-hour transformed time interval was specified using the “TINTERVAL” statement to account for unequally-spaced intervals due to missing data and random sampling within blocks. This procedure creates a new time variable (measured in hours since first assessment in this study) and inserts based on the defined metric missing data records when no observation is present [for a detailed overview of this approach see (63)]. Missing data in DSEM is handled using a Kalman filter approach. Due to this procedure, all observations can be used in the analyses and a constant interpretation of lagged relations is maintained (66). Given that treating covariates as exogenous variables in time-series settings may yield biased estimates (65), autoregressive effects of covariates were included in both RDSEM and DSEM models. Bayesian linear regressions were used to predict continuous variables, such as NSSI thoughts, whereas Bayesian probit regression was used to predict the occurrence of NSSI behavior, which was modeled as a categorical variable (present/absent). Each model was specified using random intercepts with all other within-level parameters fixed, and was estimated using a minimum of 2,500 iterations with a thinning parameter of 20. Model convergence was ensured by checking that the potential scale reduction was close to 1 and trace plots did not contain trends, spikes, or other irregularities.

## Results

### Preliminary Descriptive and Variability Analyses

**Figure 3:**
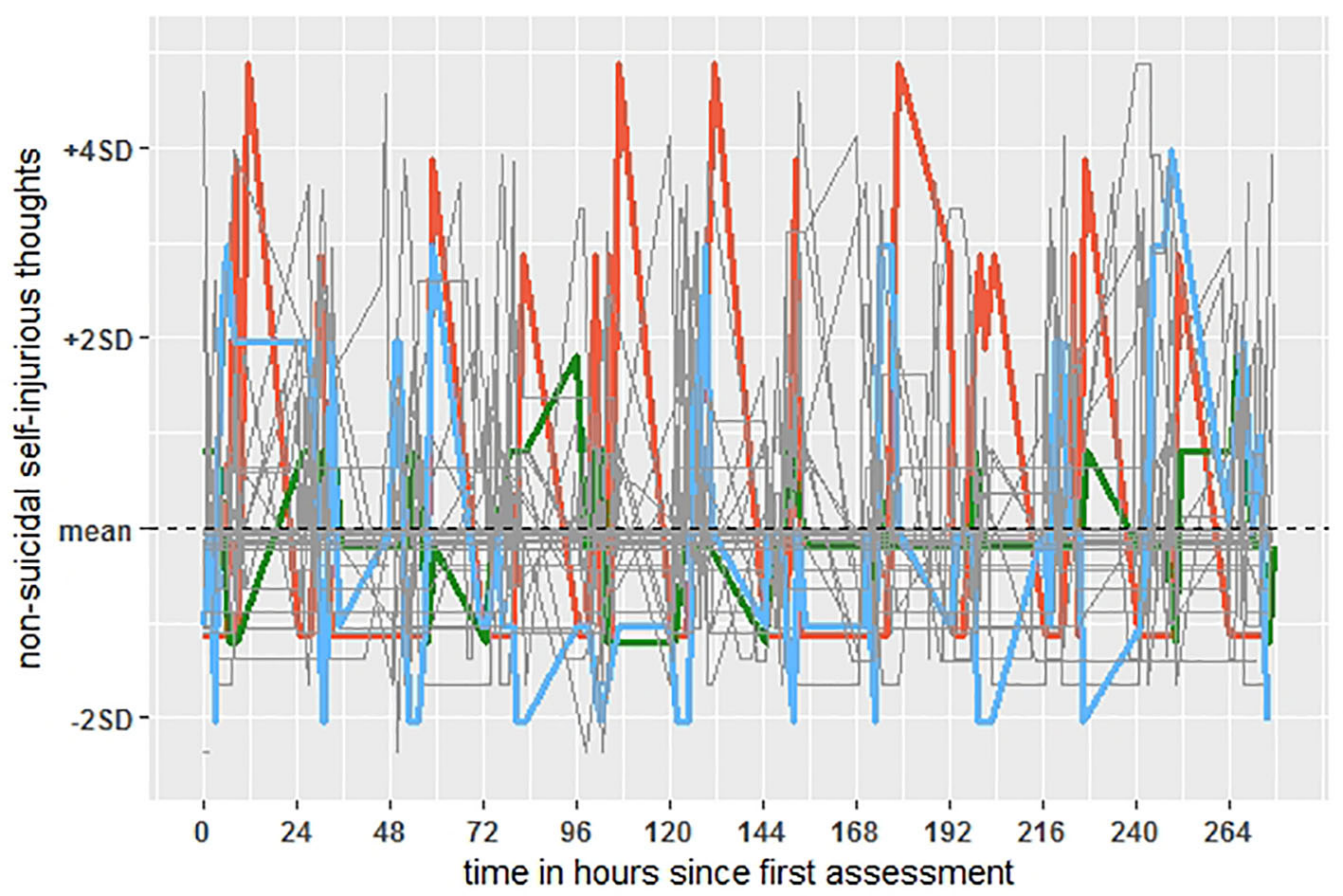
Times series plot of non-suicidal self-injurious thoughts during the 12-day assessment period. Values are person-mean centered (comparing each's participant's hourly level of non-suicidal self-injurious thoughts to that individual's overall average across time; dashed line). The colored lines represent three randomly selected participants to illustrate within-person variability on an hourly basis.

During the 12-day experience sampling protocol, 591 NSSI thoughts (i.e., score higher than 0; mean intensity = 0.72; SD = 1.48) were reported. Among those reporting NSSI thoughts (90%), on average 21.9 (SD = 21.4; median = 16.0; range 1–70) NSSI thoughts were reported. Of the sample, 53.3% of the participants engaged in NSSI, with an average of 10.4 episodes during the 12-day experience sampling protocol (SD = 10.7; median = 6.0; range 1–37). In total, 270 NSSI behaviors were recorded across 167 assessments (7.5% of all sampled time points). **Table 2** presents the descriptive and variability statistics for all within and between-person variables. These findings show that approximately half of the variability in negative affect and NSSI thoughts is due to within-person variance (vs. between person-variance). **Figure 3** illustrates the within-person variability of NSSI thoughts on an hourly basis for participants. Although self-efficacy to resist NSSI varied more between than within individuals, positive affect showed considerably more variation at the within-person level.

**Table 2:** Descriptive and variability statistics of negative affect, positive affect, self-efficacy to resist NSSI, and NSSI thoughts and behaviors during 12-day experience sampling protocol.

Within-person variables	M/N	SD/%	Range	Total variance^a^	ICC^b^	95% CI
NSSI thoughts	0.72	1.48	0–6	2.38	.51	.38-.65
Negative affect	1.74	1.19	0–6	1.52	.46	.34-.61
Positive affect	2.94	1.28	0–6	1.73	.33	.23-.47
Self-efficacy to resist NSSI next hours	4.79	1.74	0–6	3.34	.70	.59-.80
Number of assessments NSSI behavior reported	167	7.52	0–1	–	–	–
**Between-person variables**	**M**	**SD**	**Range**	–	–	–
Trait negative affect	29.33	7.88	5–50			
Trait positive affect	30.13	5.21	5–50			
Self-efficacy to resist NSSI next 2 weeks	31.93	10.73	6–60			
Anxiety symptoms past week	13.60	3.66	7–28			
Depressive symptoms past week	14.53	5.24	7–28			

a Total variance represents the sum of variance within individuals across time (i.e., within-person variance) and variance in within-person means across individuals (i.e., between-person variance).

b The ICC represents the proportion of the total variance that is accounted for by between-person variance.

NSSI, non-suicidal self-injury; ICC, intraclass correlation; 95% CI, credibility interval; M, mean; SD, standard deviation; N, total number.

### Contemporaneous Associations Between Affect, Self-Efficacy to Resist NSSI, and NSSI Thoughts (Objective 1a)

We first investigated how variables were contemporaneously associated within the same time frame after partialing out temporal associations (**Table 3**). In univariate analyses, negative affect was significantly positively associated with NSSI thoughts, whereas positive affect and self-efficacy to resist NSSI were each negatively associated with NSSI thoughts. After controlling for shared variance within a multivariate modeling framework, each factor remained significantly associated, although weaker, with NSSI thoughts. Together, these contemporaneous associations explained 41% of the within-person variance of NSSI thoughts in this sample.

**Table 3:** Contemporaneous (moment-to-moment) associations between affective states, self-efficacy to resist NSSI, and non-suicidal self-injurious thoughts.

	Univariate analyses^a^		Full multivariate analyses^b^	
	B (SD)	95% CI	B (SD)	95% CI
Contemporaneous within-person associations				
Negative affect *t*	**0.48 (0.02)**	**0.43**; **0.53**	**0.28 (0.03)**	**0.22**; **0.33**
Positive affect *t*	−**0.33 (0.02)**	−**0.37**; −**0.29**	−**0.05 (0.02)**	−**0.09**; −**0.00**
Self-efficacy to resist NSSI *t*	−**0.58 (0.02)**	−**0.61**; −**0.53**	−**0.48 (0.02)**	−**0.52**; −**0.45**

^a^Univariate analyses are based on separate multilevel regression models for each row, with the variable in the row as predictor. ^b^The multivariate model includes all within-person level variables in one multilevel regression model (cf. **Figure 1A**).

B, median unstandardized point estimate; SD, posterior standard deviation; CI, credibility interval.

Bolded cells indicate that there is a 95% probability that the true population value is not null.

### Fluctuations in Affective States and Self-Efficacy to Resist NSSI as Real-Time Predictors of NSSI Thoughts (Objective 1b)

We constructed temporal models to determine the utility of negative affect, positive affect, and self-efficacy to resist NSSI as predictors of short-term change in NSSI thoughts (**Table 4**). Higher-than-usual negative affect at *t* − 1 positively predicted NSSI thoughts at *t*, whereas higher-than-usual positive affect and self-efficacy to resist NSSI at *t* − 1 negatively predicted NSSI thoughts at *t*. In a next step, we evaluated the unique value of these factors in a multivariate prediction model that simultaneously included all cross-regressive parameters at *t* − 1. As can be seen in **Table 4**, higher-than-usual positive affect at *t* − 1 became non-significant in predicting short-term change in NSSI thoughts at *t*. Fluctuations in negative affect and self-efficacy to resist NSSI at *t* − 1 remained significantly predictive of short-term change in NSSI thoughts at *t*. Together, these temporal associations explained 18% of the variance of NSSI thoughts within persons, across time.

**Table 4:** Temporal within-person associations between affective states, self-efficacy to resist NSSI, and non-suicidal self-injurious thoughts.

	Analyses controlling NSSI thoughts at *t* – 1 h^a^		Full multivariate analyses^b^	
	B (SD)	95% CI	B (SD)	95% CI
Temporal within-person associations				
NSSI thoughts *t* – 1 h	**0.47 (0.02)**	**0.43**; **0.52**	**0.26 (0.03)**	**0.20**; **0.33**
Negative affect *t* – 1 h	**0.17 (0.03)**	**0.11**; **0.23**	**0.09 (0.04)**	**0.02; 0.17**
Positive affect *t* – 1 h	**–0.10 (0.02)**	**–0.15**; −**0.05**	0.00 (0.03)	–0.05; 0.06
Self-efficacy to resist NSSI *t* – 1 h	**–0.23 (0.03)**	**–0.30**; −**0.17**	**–0.18 (0.03)**	**–0.24**; −**0.12**

^a^Analyses are based on separate multilevel regression models for each row, with the variable in the row as predictor and controlling the autoregressive parameter of NSSI thoughts. ^b^The multivariate model includes all within-person variables in one multilevel regression model (cf. **Figure 1B**).

B, median unstandardized point estimate; SD, posterior standard deviation; CI, credibility interval.

Bolded cells indicate that there is a 95% probability that the true population value is not null.

### Fluctuations in Affective States and Self-Efficacy to Resist NSSI as Real-Time Predictors of NSSI Behavior (Objective 2)

To investigate the utility of fluctuations in affective states and self-efficacy to resist NSSI in predicting the occurrence of NSSI behavior beyond NSSI thoughts, we compared temporal models that included the autoregressive parameter of NSSI behavior to models that controlled the cross-regressive parameter of NSSI thoughts (**Table 5**). As expected, a very similar pattern of results as above was observed in models that included the lagged version of NSSI behavior. Higher-than-usual negative affect at *t* − 1 was significantly positively predictive of the occurrence of NSSI behavior, whereas higher-than-usual positive affect and self-efficacy to resist NSSI at *t* − 1 were significantly negatively predictive of NSSI behavior in the next time interval. However, when we controlled the cross-regressive parameter of NSSI thoughts at *t* − 1, fluctuations in negative affect and positive affect at *t* − 1 both became non-significant predictors of NSSI behavior at *t*. In contrast, higher-than-usual belief in one's ability to resist NSSI at *t* − 1 remained significantly predictive of a lower probability of NSSI behavior at *t*.

**Table 5:** Temporal within-person associations between affective states, self-efficacy to resist NSSI, and non-suicidal self-injury.

	Analyses controlling NSSI behavior *t* – 1 h^a^		Analyses controlling NSSI thoughts *t* – 1 h^b^	
	B (SD)	95% CI	B (SD)	95% CI
Temporal within-person associations				
NSSI behavior *t* – 1 h	**0.31 (0.10)**	**0.07**; **0.49**	0.04 (0.14)	−0.23; 0.28
NSSI thoughts *t* – 1 h	**0.32 (0.07)**	**0.20**; **0.45**	**0.32 (0.05)**	**0.21**; **0.43**
Negative affect *t* – 1 h	**0.26 (0.08)**	**0.12**; **0.41**	0.14 (0.10)	−0.06; 0.33
Positive affect *t* – 1 h	−**0.19 (0.06)**	−**0.32**; −**0.09**	−0.12 (0.07)	−0.26; 0.03
Self-efficacy to resist NSSI *t* – 1 h	−**0.43 (0.08)**	−**0.58**; −**0.29**	−**0.33 (0.08)**	−**0.49**; −**0.19**

a Analyses are based on separate multilevel regression analyses for each risk and protective factor, with the factor in the row the predictor and controlling the autoregressive parameter of NSSI behavior.

b Analyses are based on multilevel regression analyses for each risk and protective factor, with the variable in the row as predictor and controlling the cross-regressive parameter of NSSI thoughts (cf. **Figure 2**).

B, median unstandardized point estimate; SD, posterior standard deviation; CI, credibility interval.

Bolded cells indicate that there is a 95% probability that the true population value is not null.

Next, we evaluated whether specific emotions, rather than affective composite scores, hold incremental predictive value in predicting NSSI behavior (**Table 6**). All assessed negative emotions at *t* − 1 (except for feeling irritated) were predictive of NSSI behavior at *t* in models including the lagged version of NSSI behavior. However, when controlling the cross-regressive parameter of NSSI thoughts at *t* − 1, again, all negative emotions at *t* − 1 became non-significant in predicting NSSI behavior at *t*. Conversely, all assessed positive emotions at *t* − 1 were consistently negatively predictive of NSSI behavior at *t* in models including the lagged version of NSSI behavior at *t* − 1. However when controlling the cross-regressive parameter of NSSI thoughts at *t* − 1, the feeling “relaxed” at *t* − 1 remained negatively predictive of NSSI behavior at *t*.

**Table 6:** Temporal within-person associations between specific emotions and non-suicidal self-injury.

	Analyses controlling NSSI behavior *t* – 1 h^a^		Analyses controlling NSSI thoughts *t* – 1 h^b^	
Temporal within-person associations	B (SD)	95% CI	B (SD)	95% CI
Negative emotions high-arousal				
Anxious *t* – 1 h	**0.21 (0.07)**	**0.08**; **0.35**	0.10 (0.08)	−0.05; 0.27
Irritated *t* – 1 h	0.09 (0.06)	−0.03; 0.20	0.06 (0.06)	−0.07; 0.18
Stressed *t* – 1 h	**0.13 (0.05)**	**0.03**; **0.23**	0.09 (0.06)	−0.02; 0.21
Negative emotions low-arousal				
Sad *t* – 1 h	**0.13 (0.05)**	**0.04**; **0.23**	0.08 (0.06)	−0.04; 0.19
Hopeless *t* – 1 h	**0.11 (0.05)**	**0.01**; **0.21**	0.02 (0.06)	−0.11; 0.14
Insecure *t* – 1 h	**0.17 (0.07)**	**0.05**; **0.31**	0.08 (0.07)	−0.05; 0.22
Positive emotions high-arousal				
Cheerful *t* – 1 h	−**0.17 (0.06)**	−**0.28**; −**0.07**	−0.12 (0.06)	−0.24; 0.00
Excited *t* – 1 h	−**0.13 (0.05)**	−**0.23**; −**0.04**	−0.05 (0.06)	−0.18; 0.07
Positive emotions low-arousal				
Satisfied *t* – 1 h	−**0.11 (0.05)**	−**0.21**; −**0.01**	−0.05 (0.06)	−0.17; 0.08
Relaxed *t* – 1 h	−**0.19 (0.06)**	−**0.30**; −**0.08**	−**0.14 (0.07)**	−**0.27**; −**0.01**

a Analyses are based on separate multilevel regression analyses for each risk and protective factor, with the factor in the row predictor and controlling the autoregressive parameter of NSSI behavior.

b Analyses are based on multilevel regression analyses for each risk and protective factor, with the variable in the row as predictor and controlling the cross-regressive parameter of NSSI thoughts (cf. **Figure 2**).

B, median unstandardized point estimate; SD, posterior standard deviation; CI, credibility Interval.

Bolded cells indicate that there is a 95% probability that the true population value is not null.

### Trait Affect, Self-Efficacy to Resist NSSI, and Anxiety and Depressive Symptoms as Predictors of NSSI Thoughts and NSSI Behaviors (Objective 3)

Finally, we examined the utility of baseline measures of trait affect, self-efficacy to resist NSSI, and past-week anxiety and depressive symptoms as between-person predictors of NSSI thoughts and NSSI behavior (**Supplementary Materials**). This revealed that individuals with lower mean scores on trait positive affect (β = −0.09, 95% CI = −0.16; −0.02) and higher mean scores on past week depressive symptoms (β = 0.11, 95% CI = 0.04; 0.18) reported higher mean levels of NSSI thoughts across the 12-day experience sampling protocol. Yet, only depressive symptoms uniquely predicted a higher mean level of NSSI thoughts across time (β = 0.11, 95% CI = 0.01; 0.22). In contrast, self-efficacy to resist NSSI at baseline was the only factor that significantly predicted engagement in NSSI behavior during the 12-day experience sampling protocol (β = −0.06, 95% CI = −0.15; −0.00).

## Discussion

Obtaining a better understanding of the factors that determine when individuals at high risk are most likely to contemplate, or engage in, NSSI behavior represents a challenging but critical research frontier (35, 67). To this end, using smartphone-based assessment of young adults who frequently self-injure, the present study provides a preliminary investigation into the extent to which affective states and self-efficacy to resist NSSI are real-time predictors of NSSI thoughts and behaviors. To our knowledge, this is the first experience sampling study to differentiate between the process of experiencing NSSI thoughts and engaging in NSSI behavior. Results suggest that affective fluctuations (especially negative affect) may be more useful in predicting NSSI thoughts than behavior per se, and point to the role of cognitive factors (i.e., belief in one's ability to resist NSSI) in preventing NSSI behavior among people experiencing NSSI thoughts.

NSSI thoughts varied considerably across hours, illustrating the need for intensive monitoring to capture these fluctuations in daily life. The first aim of the study was to identify real-time factors that explain variability in NSSI thoughts. Consistent with previous work (40, 41, 48), higher-than-usual negative affect co-occurred with NSSI thoughts and uniquely predicted a stronger intensity of NSSI thoughts from one observation window to the next. The latter provides further evidence that increased negative affect is a robust short-term risk factor for NSSI thoughts. In contrast, while lower-than-usual positive affect was negatively associated with NSSI thoughts, this association did not transcend uniquely across time periods. There are two explanations for this: a) positive affect is only relevant in identifying NSSI thoughts as they occur, or b) positive affect also acts as a buffer against NSSI thoughts, but this protective effect occurs on a shorter timescale than the hourly scale used in this study. In line with the latter, we observed substantial within-person variability [intraclass correlation (ICC) = 0.33] in positive affect from hour to hour. However, future research with even greater temporal resolution is needed to rule out one of these explanations. Finally, we found evidence that individuals were less likely to consider NSSI when they had high momentary belief in their ability to resist NSSI, which, in turn, prospectively predicted a lower intensity of NSSI thoughts one hour later.

Importantly, associations between affective states and NSSI thoughts were considerably weaker in temporal than contemporaneous models. Although the temporal precedence of associations cannot be determined in contemporaneous models (i.e., whether affect changes NSSI thoughts, or vice versa), researchers have advocated that contemporaneous relationships, which represent a snapshot in time, may uncover fast-moving causal processes (54). Given the time frame of measurement in this study, this likely suggests that the connection between momentary affect and the manifestation of NSSI thoughts is a fast occurring process that operates within seconds and minutes rather than hours. Again, this implies that better understanding the time frame of these relationships represents an important avenue for future research, as this will provide unique insight into effects that may unfold across shorter and/or longer time intervals.

The second aim of the study was to evaluate the extent to which fluctuations in affective states and self-efficacy to resist NSSI predict NSSI behavior beyond the effect of NSSI thoughts. In line with previous work (39–41), we found that fluctuations in negative affect prospectively predicted NSSI behavior when NSSI thoughts were not accounted for. When accounting for NSSI thoughts, however, negative affect was no longer significantly predictive of NSSI behavior. Following an ideation-to-action framework (45–47), we do not believe this pattern of findings to indicate that negative affect is unimportant in the manifestation of NSSI behavior—indeed it leads people at high risk to more intensively contemplate engaging in NSSI—but only that it will not exert an additional effect beyond intensity of thoughts in determining whether someone will progress and engage in NSSI. We found similar findings for positive affect: higher-than usual positive affect was not uniquely predictive of a lower probability of engaging in NSSI behavior when taking into consideration NSSI thoughts. Further analyses showed similar findings for all but one emotion (i.e., feeling relaxed), which reflects—relative to feeling satisfied—an absence of arousal within the low positive valence quadrant (61, 68). Although caution is needed interpreting this finding, it suggests that focusing on the down-regulation of physiological hyper-arousal (69, 70), when thoughts of NSSI occur, may be one useful strategy to interrupt the transition to NSSI behavior. Taken together, these findings provide preliminary evidence that affective states may be unique real-time predictors of NSSI thoughts but not behavior.

If replicated, the implications are far-reaching as it would reflect the necessity of treating the development of thoughts and the subsequent transition from NSSI thoughts to behavior as separate processes that may come with separate sets of predictors. Making the distinction between NSSI thoughts/behaviors may not only be important from a theoretical, but also from a clinical viewpoint. Researchers observed that it typically takes people who self-injure between 1 and 30 minutes to transition from thoughts to behavior (42, 71). This implies that, in most instances, there will be a brief window of opportunity to intervene and interrupt the transition to behavioral action. Ecological momentary interventions using mobile devices might have particular merit in this context (22, 72, 73), as these can be delivered when people report experiencing NSSI thoughts, and facilitate relapse prevention techniques. In line with the Cognitive-Emotional Model of NSSI (23), we found evidence that low self-efficacy to resist NSSI may be particularly relevant in identifying high-risk situations among people experiencing NSSI thoughts.

The third aim of the study was to investigate population-level predictors at the between-person level. In line with findings in suicide research (47, 49, 50) and the Cognitive-Emotional Model of NSSI (23), people with higher levels of depressive symptoms at baseline reported more intense thoughts over the course of the study, but only low self-efficacy to resist NSSI in the next two weeks explained who, in our student sample, engaged in NSSI. This is consistent with the concept of capability for suicide (74), which specifies that a person must hold beliefs about their ability to self-injure (i.e., low self-efficacy to resist) in order to act on self-injurious thoughts. In sum, these findings provide novel evidence for the clinical utility of NSSI-related cognitions in determining relative risk of future NSSI behavior, and suggest that boosting self-efficacy to resist NSSI might be an important step in equipping people who self-injure with the confidence to handle high risk aversive emotional situations in everyday life.

### Limitations and Future Research Directions

Several limitations should be considered in interpreting the findings of this study. First, and foremost, as this sample comprised 30 (mostly female) young adults, replication is warranted in larger samples including more males. Second, and relatedly, the generalizability of the findings to clinical samples is unclear and should be studied. It might be that clinical samples show stronger temporal relationships between affective states and NSSI thoughts and behaviors. The current findings should, therefore, be considered as preliminary. In fact, a major future research avenue will be to allow subject-specific effects (for which the current sample was too small) to clarify how these within-person relationships differ between people, as a function of person-level features, such as diagnostic status, gender, personality traits, and experienced life events. Third, all participants within the sample had already engaged in NSSI. This is in contrast to the majority of ideation-to-action research on suicidal thoughts and behaviors, where ideation is considered only in the absence of behavior and separate groups of individuals with ideation and those with behavior are compared. It is possible that factors predicting NSSI thoughts may differ between individuals who have and have not already engaged in NSSI behavior. Contemporary ideation-to-action models of suicide have not explicitly considered factors associated with ideation among individuals who have already engaged in suicidal behavior (45, 46, 74, 75). Consequently, a fruitful direction for future research could be to compare ideation-to-action pathways between those who have and have not already engaged in NSSI. Fourth, while the experience sampling protocol we implemented is among the most longitudinally intensive studies conducted thus far (assessments every 90 minutes), this did not allow us to track dynamic processes that happen in the moments that lead up to NSSI. To address this shortcoming, future experience sampling studies could incorporate burst assessments (i.e., multiple beeps over shorter time periods) when individuals report NSSI thoughts. Given that NSSI typically occurs within a narrow time frame following NSSI thoughts (42, 71), such studies would also provide a unique opportunity to clarify the immediate affective-cognitive consequences of engaging in NSSI.

Fifth, although experience sampling reduces recall bias, it still relies on self-report and the ability of participants to accurately describe their thoughts, feelings, and behaviors. Future studies may want to investigate if incorporating wearable devices that detect information about people's movement and activity and psychophysiology (e.g., electro-dermal activity and heartrate variability) could augment the short-term prediction of NSSI thoughts and behavior beyond self-report. Use of wearable technology for these purposes is already emerging in suicide research (76, 77). Sixth, although overall compliance was high, considering the intensive sampling protocol, on average participants failed to respond to one in five prompts, and it is unclear to what extent this may have impacted the results. Finally, to reduce participant burden, we decided to operationalize NSSI thoughts using a single item similar to previous studies (42). Building upon these findings, future studies may want to evaluate different qualitative aspects relating to NSSI thoughts [i.e., intensity, duration, controllability; (71, 78)], and explore whether meaningful patterns can be identified in relationship to risk for NSSI behavior. In suicide research, for instance, scholars have identified different phenotypes of suicidal thinking, and were able to associate a thought profile characterized by severe persistent suicidal thoughts to a recent suicide attempt (79).

## Conclusion

The present study provides novel evidence that affective fluctuations may be more central to the prediction of NSSI thoughts than NSSI behavior, and suggests that perceiving oneself to be able to resist NSSI, might be key in determining risk of NSSI behavior among people experiencing NSSI thoughts. We believe these findings illustrate the merit of carefully delineating between the processes of developing thoughts and making the transition to behavior, and we hope it encourages researchers to further investigate the relative importance of momentary factors for the different stages towards engagement in NSSI.

## Data Availability Statement

The datasets generated for this study are available on request to the corresponding author.

## Ethics Statement

The studies involving human participants were reviewed and approved by the Social and Societal Ethics Committee (KU Leuven). The patients/participants provided their written informed consent to participate in this study.

## Author Contributions

GK: study design, data collection, data analysis, interpretation of results, writing initial drafts of the manuscript, and critical revision for important intellectual content. PH: study design, interpretation of results, and critical revision for important intellectual content. MN: critical revision for important intellectual content. MB: study design and critical revision for important intellectual content. OK: study design and critical revision for important intellectual content. IM-G: study design and critical revision for important intellectual content. RB: critical revision for important intellectual content. LC: study design, data-collection, interpretation of results, critical revision for important intellectual content, and supervision of all aspects of the study. All authors approved the final version of the manuscript.

## Funding

This research was supported by grants from the Research Foundation Flanders [1114717N (GK), 1114719N (GK)], and Curtin University [CIPRS/HSFIRS (GK)]. The funding sources had no role in the design and conduct of the study; collection, management, analysis, and interpretation of the data; preparation, review, or approval of the manuscript; and decision to submit the manuscript for publication.

## Conflict of Interest

The authors declare that the research was conducted in the absence of any commercial or financial relationships that could be construed as a potential conflict of interest.
