# Supplementary material for: Fluctuations in Affective States and Self-Efficacy to Resist Non-Suicidal Self-Injury as Real-Time Predictors of Non-Suicidal Self-Injurious Thoughts and Behaviors
Source: Front Psychiatry. 2020 Mar 20;11:214. doi: 10.3389/fpsyt.2020.00214 (PMC7099647; doi:10.3389/fpsyt.2020.00214)
Supplement: Supplementary file 1 [file Table_1.docx]

**Supplementary Table 1**

Temporal between-person associations between trait affect, self-efficacy to resist NSSI, anxious and depressive symptoms and NSSI thoughts during the 12-day experience samling protocol

|  | **Univariate analyses^a^** | | **Full multivariate analyses^b^** | |
| --- | --- | --- | --- | --- |
|  | Β (SD) | 95% CI | Β (SD) | 95% CI |
| **Temporal between-person associations** |  |  |  |  |
| Trait negative affect | 0.02 (0.03) | -0.04; 0.07 | -0.02 (0.04) | -0.09; 0.05 |
| Trait positive affect | **-0.09 (0.04)** | **-0.16; -0.02** | -0.04 (0.04) | -0.13; 0.04 |
| Self-efficacy to resist NSSI during study | -0.03 (0.02) | -0.07; 0.01 | -0.01 (0.02) | -0.05; 0.03 |
| Anxiety symptoms past week | 0.04 (0.06) | -0.07; 0.15 | -0.04 (0.07) | -0.19; 0.11 |
| Depressive symptoms past week | **0.11 (0.03)** | **0.04; 0.18** | **0.11 (0.05)** | **0.01; 0.22** |

Note: ^a^ Analyses are based on separate multilevel regression models for each row, with the variable in the row as between-person predictor and controlling the autoregressive parameter of NSSI thoughts at the within-person level (not shown here). ^b^ The multivariate model includes all between-person level variables in one multilevel regression model. Β = median unstandardized point estimate; SD = posterior standard deviation; CI = Credibility Interval. Bolded cells indicate that there is a 95% probability that the true population value is not-null.
