# Supplementary material for: Fluctuations in Affective States and Self-Efficacy to Resist Non-Suicidal Self-Injury as Real-Time Predictors of Non-Suicidal Self-Injurious Thoughts and Behaviors
Source: Front Psychiatry. 2020 Mar 20;11:214. doi: 10.3389/fpsyt.2020.00214 (PMC7099647; doi:10.3389/fpsyt.2020.00214)
Supplement: Supplementary file 2 [file Table_2.docx]

**Supplementary Table 2**

Temporal between-person associations between trait affect, self-efficacy to resist NSSI, anxious and depressive symptoms and NSSI during the 12-day experience sampling protocol

|  | **Univariate analyses** | |
| --- | --- | --- |
|  | Β (SD) | 95% CI |
| **Temporal between-person associations** |  |  |
| Trait negative affect | -0.01 (0.05) | -0.11; 0.08 |
| Trait positive affect | -0.07 (0.09) | -0.26; 0.07 |
| Self-efficacy to resist NSSI at baseline | **-0.06 (0.04)** | **-0.15; -0.00** |
| Anxiety symptoms past week | 0.05 (0.10) | -0.13; 0.27 |
| Depressive symptoms past week | 0.09 (0.07) | -0.03; 0.25 |

Note: Analyses are based on separate multilevel regression models for each row, with the variable in the row as between-person predictor and controlling the autoregressive parameter of NSSI behavior at the within-person level (not shown here). Β = median unstandardized point estimate; SD = posterior standard deviation; CI = Credibility Interval. Bolded cells indicate that there is a 95% probability that the true population value is not-null.
